# Ameliorative effects of *Spirulina platensis* against lead-induced nephrotoxicity in newborn rats: Modulation of oxidative stress and histopathological changes

**DOI:** 10.17179/excli2017-1016

**Published:** 2018-03-02

**Authors:** Manel Gargouri, Ahlem Soussi, Amel Akrouti, Christian Magné, Abdelfattah El Feki

**Affiliations:** 1 Laboratory of Animal Ecophysiology, Faculty of Sciences, Sfax University, Tunisia; 2 EA2219 Geoarchitecture, University of Brest Cedex 3, France

**Keywords:** antioxidant activities, DNA and mRNA damage, nephrotoxicity, neonate rats, oxidative stress, Spirulina supplementation

## Abstract

Our experimental work was aimed at evaluating the safety and protective effects of dietary *spirulina *supplementation on the kidney of newborn rats, the offspring of lead contaminated lactating mothers. Female rats were randomly divided into four groups: group I (control) was given a normal diet, group II (positive control, S) received a diet enriched with *spirulina*, group III received only lead through drinking water (Pb), and group IV received both a diet enriched with *spirulina* and lead contaminated water (S Pb). The treatment of pregnant rats with lead administrated in drinking water, from the 5^th^ day of pregnancy until day 14 after delivery, induced an increased level of renal lipid peroxidation, protein carbonyl, hydrogen peroxide and advanced oxidation protein product, a decreased renal content of glutathione and antioxidant enzyme activities such as superoxide dismutase, catalase and glutathione peroxidase in newborns. A statistically significant increase of renal DNA, mRNA, hematological parameters as well as in plasma urea and creatinine serum levels and lactate dehydrogenase was seen in pups, while those of uric acid declined. Interestingly, these biochemical modifications were accompanied by a significant decrease of lactate dehydrogenase in kidney, plasma alkaline phosphatase and gamma glutamyl-transpeptidase levels, urinary levels of creatinine and urea. Conversely, supplementation of lead-treated mother's with *spirulina* alleviated hematotoxicity induced by lead as evidenced, by restoring the biochemical markers cited above to near normal levels. Nevertheless, the distorted histoarchitecture in rat kidney attenuated following spirulina supplementation. It can be then concluded that spirulina is an important protective source against kidney impairments.

## Introduction

Spirulina (*Arthrospira platensis *Gomont) is a filamentous microalga which grows in fresh water and has a simple structure but a complex composition. Early studies were mainly focused on the nutritional value of spirulina with its high protein content (up to 70 %) and its active constituents such as vitamins (ß-carotene), minerals, tocopherols and phenolic acids (Khan et al., 2005[42]). C-phycocyanin, one of the major biliproteins, therefore we need to prove the alga strong antioxidant and anti-inflammatory activities (Alvarenga et al., 2011[8]; Abdel-Daim, 2014[2]). Spirulina extracts have been recently investigated *in vitro *and* in vivo*. They are considered as a food additive (Abdel-Daim et al., 2013[3]). Moreover, this alga possesses many biological properties, like prevention of anemia (Hemalatha et al., 2012[37]), inhibition of herpes infection (Ferreira-Hermosillo et al., 2011[29]), reduction of HIV replication velocity (Ayehunie et al., 2008[14]), stimulation of antibody production, as well as hepatoprotective, neuroprotective, and antigenotoxic activities (Reddy et al., 2000[53]; Gargouri et al., 2012[33], 2016[34]). Furthermore, spirulina has been reported to have the abiblity to protect many organs from heavy metal-induced toxicity (Paniagua-Castro et al., 2011[51]; El-Desoky et al., 2013[25]).

A number of metals are required at low doses in living organisms. Nevertheless, their extensive use has been accompanied by high concentrations in the environment (atmosphere, water, ground) and plants, with potential toxic effects on human. This results from either agricultural or industrial production or through accidental or deliberate misuse (Janicka et al., 2015[40]). For many years, lead has been recognized as a highly toxic metal affecting every body organ in humans and animals at all ages, especially young children (Markowitz, 2011[46]; Ibrahim et al., 2012[38]). Furthermore, toxicological studies have shown that following extensive oral absorption, lead underwent a wide distribution in the body and excretion predominantly via urine, where numerous metabolites have been identified (Abdel-Moneim, 2015[5]).

In general, the mammalian kidney has an important role in the body homeostasis, through, the excretion of metabolic wastes and the regulation of extracellular fluid volume, electrolyte composition, and acid-base balance (Schnellmann, 2008[58]). In addition, kidney is particularly suceptible to lead, causing proximal tubular malfunction or irreversible nephropathy depending on the exposure type (Conterato et al., 2007[21]). The biotransformation of lead results in the production of reactive oxygen or nitrogen species (ROS and RNS, respectively) which are responsible for oxidative injury (Jacobs and Marnett, 2010[39]). The proposed mechanisms of lead toxicity involve fundamental biochemical processes, including the inhibition of metabolic activities, the interaction with proteins, DNA alteration (Avery, 2011[13]), as well as the reduction of the antioxidant defences (Singh et al., 2013[60]).

Although many reports have been published about the sub-acute toxicity of lead (EFSA, 2013[23]; Tsai et al., 2016[63]), few of them have studied the natural products that may overcome this toxicity, and the mechanism of their ameliorative actions. We hypothesize that co-administration of natural products can minimise lead toxic effects. Therefore, the present study was conducted to assess some biochemical parameters, antioxidant status and histopathological alterations in the kidney of lead-exposed rat neonates and evaluate the antagonistic role of spirulina against subacute lead-induced nephrotoxicity in offspring.

## Methods and Materials

### Materials

Lead acetate Pb(C_2_H_3_O_2_)_2_ was obtained from SD Fine Chemicals. Other products such as 5,5'-dithio-bis2-nitrobenzoic acid (DTNB), glutathione (oxidized and reduced form) were purchased from Sigma. All other chemicals and reagents were of analytical grade and used as received.

### Preparation of algae

Fresh *Spirulina platensis* algae were identified by Prof Y Krichen (National Agronomic Institute of Tunisia). The algal powder was mixed with standard food pellets in distilled water to get 5 % (500 mg/kg of diet) supplementation (Gargouri et al., 2012[33]). These dietary seeds were used throughout this experiment.

### Ethics statement

The experimental procedures, animal handling, sampling, and scarification were done according to the Natural Health Institute of Health Guidelines for Animal Care and approved by the “Institute Ethical Committee Guidelines” Council of European Communities (1986[22]), Sciences Faculty of Sfax (n° 1204).

### Animal diet and tissue preparation

Adult Wistar Albino rats aged 2 months (weighing 170-180 g) were used. Animals were kept in cages under standards laboratory conditions (21±1 °C, 10 h/14 h light/dark cycle and 40±10 % of humidity). Commercial food pellets (SNA, Sfax, Tunisia) and distilled water were given ad libitum.

One week of acclimatization, male and virgin female rats were housed by pairs overnight in each cage for mating. The zero day of pregnancy was determined by the presence of the vaginal plug in pregnant female rats. These latter were housed individually in plastic cages.

The 32 pregnant female rats were randomly allocated into the following four groups of 8 each:

**Group 1** (Controls): rats received distilled water and a normal diet.

**Group 2** (S): rats received distilled water and a diet enriched with 5 % spirulina. This dose was selected from a preliminary study (Gargouri et al., 2012[33]) ensured a beneficial protection against toxicity. A lower dose of spirulina provides less protection while higher doses are not more effective but were not lethal to rats. Some methods have been reported to determine the corresponding dose of spirulina which can be used for humans (Bisson et al., 2005[19]). 

**Group 3** (Pb): rats were given water containing 6 g/L lead acetate, resulting in an average uptake of 343.6 mg lead/kg body weight/day and a normal diet. The dose of lead used in the present work was selected on the basis of a previous study carried out by Gorbel et al. (2002[35]).

**Group 4** (S Pb): received daily drinking water containing 6 g/L lead acetate and spirulina with same dose and the same way with Group 2.

Treatments were carried from day 5 of gestation to day 14 of lactation. The day of birth was mentioned as postnatal day zero.

Within 24 h after birth, sex and weight of pups from each mother were recorded. The pups were reduced to 8 for each mother (four males and four females, if possible) to ensure lactation performance (Fisheck and Rasmussen, 1987[30]). During the experimental period, food and water intake, body weights of the animals were monitored daily to each group (Table 1(Tab. 1)). The daily average food intake was calculated as the difference between the weight of food that remained (D_1_) and the initial amount (D_2_) according to the formula:


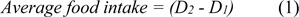


24 hours before the end of the experimental period, each animal was placed in special metabolic cages for urine collection. Urinary samples collected during 24 h cycles were recorded and centrifuged at 5000 *x g* for 5 min to eliminate the probable presence of excrements. The supernatants were stored at -20 °C for biochemical assays.

On postnatal 14^th^ day, 256 pups (control and treated rats) were weighed and sacrificed after being anesthetized with chloral hydrate solution by intra-abdominal route. Some blood samples of pups were collected into tubes containing EDTA solution used for analysis of hematological parameters. The other ones were collected into heparined tubes and centrifuged at 2500 *x g* for 15 min, to obtain plasma, which was kept at -80 °C until analysis.

Kidney tissues of control and treated pups were dissected, cleaned and weighed. Some samples were rinsed, homogenized (1:2, w/v) in Tris buffer (pH 7.4) and centrifuged at 5000 *x g* for 25 min at 4 °C. Kidney homogenate aliquots were stored at -80 <°C until biochemical analysis. The remaining kidney samples in each group were immediately fixed in 10 % buffered formalin solution for histological examination. All samples were analyzed in triplicate.

### Hematological study

Red blood cells, White (WBCs), % hematocrit, haemoglobin content, mean corpuscular volume and mean corpuscular hemoglobin concentration were analysed by an electronic automate coulter MAXM (BeckmanCoulter).

### Assays of serum markers

Plasma and urinary levels of urea, uric acid and creatinine were measured by colorimetric methods using commercial reagent kits (Ref: 20151, 20149 and 20090, respectively), purchased from Biomaghreb (Ariana, Tunis, Tunisia).

Serum levels of lactate dehydrogenase in the kidney and plasma were assayed using a commercial reagents kit (Biomaghreb, Tunisia, Ref. 20126) and were expressed in units/g in the kidney or units/L in the plasma. The absorbance of the clear supernatant was measured at 340 nm, proportional to the quantity of NADH oxidized according to the following reaction:


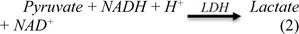


Serums levels of alkaline phosphatase (ALP) and gamma glutamyl-transpeptidase (GGT) activities were assayed using commercial diagnostic kits (Ref. 20016 and 20023, respectively), purchased from Biomaghreb.

### Biochemical determinations

#### Protein quantification

Protein content was evaluated as described by Lowry et al. (1951[45]) using bovine serum albumin as standard.

#### Estimation of TBARS, protein carbonyl, advanced oxidation protein and hydrogen peroxide generation in kidney

The level of lipid peroxide in kidney was determined spectrophotometrically according to Yagi (1976[66]). The development of pink color with the absorption at 530 nm and the TBARS values were calculated and expressed in nmol/mg protein.

Protein carbonyl (PCO) content in kidney tissue was determined using the method by Reznick and Packer (1994[54]). The absorbance using a spectrophotometer was measured at 370 nm. The carbonyl content was calculated based on the molar extinction coefficient of DNPH (ɛ= 2.2 x 10^4^ cm/M) and the results were expressed in mol/mg protein.

Advanced oxidation protein product (AOPP) levels were determined according to the method of Witko et al. (1992[65]). The absorbance was measured at 340 nm. The concentration of AOPP in each sample was calculated using the extinction coefficient of the order of 261 cm^-1^ mmol^-1^and the results were expressed in moles/mg protein.

Hydrogen peroxide (H_2_O_2_) generation in kidney tissue was assessed by Ou and Wolff method (1996[49]). The absorbance of the supernatant was recorded at 560 nm. H_2_O_2_ levels were expressed in nmol/mg protein.

#### Analysis of enzymatic and non-enzymatic activities in kidney homogenates

Superoxide dismutase (SOD) activity was determined by Beauchamp and Fridovich (1971[17]) method. The development of blue color in the reaction was measured at 560 nm. SOD activity was expressed in units per mg of protein.

Catalyze activity was assayed by H_2_O_2_ consumption, using the method of Aebi (1984[6]) and modified by Pieper et al. (1995[52]). Changes in H_2_O_2_ concentration were measured at 240 nm. The enzyme activity was expressed in mmol H_2_O_2_ consumed per min per mg of protein.

Kidney glutathione peroxidase GPx) content was measured according to Flohe and Gunzler (1984[31]). The enzyme activity was expressed in nmoles of GSH oxidized/min/mg protein.

GSH in plasma was determined by the method of Ellman (1959[26]), modified by Jollow et al. (1974[41]). The development of a yellow color in the reaction was measured at 412 nm after 10 min. Glutathione content was expressed in µg/mg of tissue.

### DNA and RNA quantifications

Total DNA and mRNA were isolated from 100 mg of kidney tissue, according to the method of Chomczynski and Sacchi (2006[20]). Each sample was measured at a wavelength of 260 nm and total DNA content was expressed in μg/g of organ (Sambrook and Russell, 2001[56]).

### Histopatological evaluation

Kidneys were fixed in Bouin's solution. They were embedded in paraffin, sectioned and stained with haematoxylin-eosin for histological investigation (Gabe, 1968[32]). Six slides were prepared from each kidney. All sections were evaluated semi-quantitatively for the degree of liver injury. All sections were estimated for the degree of tubular and glomerular injuries. Class 0: no injure; class 1: <25 % injure; class 2: 25-50 % injure; class 3: >50 % injure (Lombardi et al., 1999[44]).

### Statistical methods and evaluation

The data was reported using the statistical package program Stat Graphics plus 5.1 (stats graphics). The results expressed are average data values plus or minus standard deviation (SD). The groups were compared using one-way analysis of variance (ANOVA) followed by Fisher's protected least significant difference (FLSD) test. In all cases, differences were considered significant if *P< 0.05, **P< 0.01, or ***P< 0.001.

## Results

### Spirulina effect on general health

During the treatment period (34 days), the control and Pb-treated pregnant and lactating rats did not show either mortality or abortion. In fact, few clinical signs were noted in suckling pups, including reduced activity and increasing weakness. No significant clinical manifestations were observed in the Sp + Pb-treated rats.

In addition, mothers' exposure to lead caused a decrease in the consumption of food by 24.8 %, when compared with controls (Table 1(Tab. 1)). However, the amount of water intake increased by 30 % in Pb-group lactating rats when compared with control-group and the difference was statistically significant. Co-treatment with spirulina improved mothers' food and water consumptions which reached normal values.

Our results showed an increase in body weight about 39 %, 26 % and 30 % respectively, of pups (males and females) and pregnant and lactating mothers treated with spirulina (Table 1(Tab. 1)).

Moreover, pups from Pb group, had a markedly higher kidney weight (+82 % and 83 %, respectively) (Table 1(Tab. 1)). In S Pb group, spirulina dietary supplementation improved the parameters cited above in pups compared with those of Pb-group. Spirulina alone did not produce any significant changes of these parameters.

### Effects of spirulina on hematological parameters

Hematological parameters were measured in control and intoxicated groups (Table 2(Tab. 2)). After lead administration to pregnant and lactating mothers, our results showed a significant decrease in RBC number (29.7 %) and HB concentration (17.85 %) of 14 day-old males and 28.92 % and 21.51 % of females respectively, as compared to control (Table 2(Tab. 2)), accompanied with a significant decrease in Ht percentage (*P<0.01) *respectively. Conversely, erythrocyte parameters such as MCV, MCH, and MCHC were unchanged in both male and female newborns from lead-treated mothers.

Besides, total WBC counts were significantly increased in Pb-treated of newborns compared with those of controls (Table 2(Tab. 2)). The supplementation of spirulina to the diet of lead-intoxicated mothers ameliorated these hematological parameters of neonates.

Here again, there were no statistical differences in the parameters between spirulina and control groups (data not shown).

### Effects of spirulina on the biomarkers of nephrotoxicity

#### Creatinine, urea and uric acid levels in plasma and urine

Changes in creatinine, urea and uric acid levels in plasma and urine, in the offspring varied greatly depending on mother treatment. In fact, our results showed that lead intoxication of mothers resulted in 86.2 % and 99.5 % increases of creatinine levels in the plasma of male and female pups, respectively; but a significant reduction in urine (-69.2 % and -72.08 %, respectively), as compared to control (Table 3(Tab. 3)). However, we noted an improvement in renal biomarkers of neonates from poisoned mothers treated with spirulina.

Urea levels were respectively, higher (by 65.10 %, *P<0.01*) in plasma and lower (by 63.82 %, *P<0.01*) in urine of male offspring from lead-intoxicated mothers (Table 3(Tab. 3)). Similar observation was in female neonates (+70 % and -66.2 %, *P<0.01*, respectively, in plasma and urine). However, dietary spirulina supplementation to the lead-poisoned mothers significantly reduced the urea level in plasma and urine of newborns close to control values (*P < 0.01*).

Conversely, uric acid levels in lead treated male and female pups were respectively lower in plasma (−18.38 % and −17.60 %), and higher in urine (+25.60 % and +40.46 %) compared to those of controls. Here again, the addition of spirulina to the diet of mothers restored partially the levels of renal markers when compared to those of Pb-treated newborns. 

In addition, spirulina supplementation to the lead-poisoned mothers resulted in a significant decrease in blood urea nitrogen (BUN) levels of newborns; near-normal levels. So, spirulina alone did not produce any significant changes of these parameters.

### Plasma ALP, GGT and LDH activities and kidney LDH activity

Table 4(Tab. 4) showed that plasma ALP and GGT activities of the 14 day-old males were significantly decreased by -31.09 % and -37.75 %, respectively and of females by -25.03 % and -35.81 %, respectively, as compared to control.

In addition, activities of plasma LDH (males: +59.2 %; females: +45.62 %; (*P < 0.001*)) has increased, while the kidney homogenates decreased by -31.71 % and -26.68 % in males and females, respectively, compared with Pb-treated rats.

The supplementation of spirulina to the diet of control mothers did not produce any significant changes in the activities of newborn plasma or kidney (data not shown).

Conversely, ALP and GGT activities in the plasma and LDH in homogenates kidney of neonate increased following Pb intoxication of mothers (*P<0.05*), and dietary spirulina supplementation during gestation and lactation caused a partial decrease in plasma LDH activity, without attenuating the control values of offspring.

### Effects of spirulina on DNA and mRNA levels in the kidney

**Figure 1:**
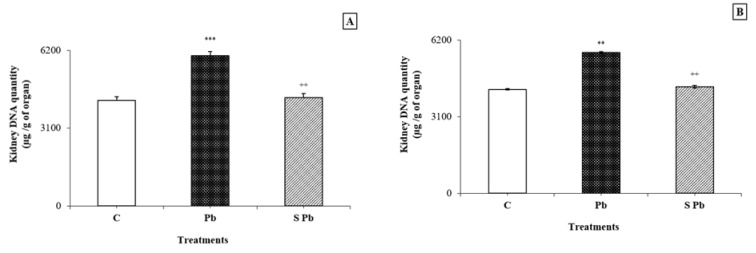
Effects of lead exposure and/or spirulina on the 14 day-old rats ((C), (Pb) and (S Pb)) on renal DNA quantity. *NB:* A: male; B: female; C: Control rats; Pb: Treated rat with lead; (S Pb): Rat treated with spirulina and intoxicated with lead. Values are expressed as means ± SD for four rats in each group. One-way analysis of variance (ANOVA) followed by Fisher's protected least significant difference (PLSD) as a post hoc test for comparison between groups: Comparison between (Pb) versus C group: *P < 0.05; **P < 0.01; ***P < 0.001. Comparison between S Pb group versus Pb group: +P < 0.05; ++P < 0.01; +++P < 0.001

**Figure 2:**
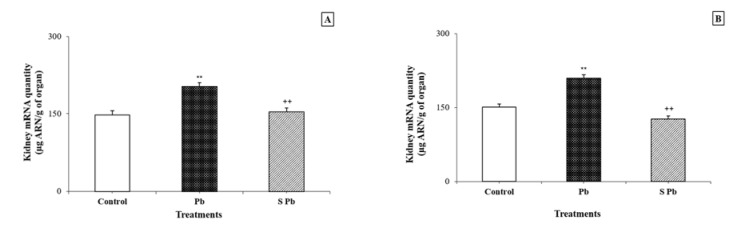
Effects of lead exposure and/or spirulina on the 14 day-old rats ((C), (Pb) and (S Pb)) on renal RNA quantity. *NB:* A: male; B: female; C: Control rats; Pb: Treated rat with lead; (S Pb): Rat treated with spirulina and intoxicated with lead. Values are expressed as means ± SD for four rats in each group. One-way analysis of variance (ANOVA) followed by Fisher's protected least significant difference (PLSD) as a post hoc test for comparison between groups: Comparison between (Pb) versus C group: *P < 0.05; **P < 0.01; ***P < 0.001. Comparison between S Pb group versus Pb group: +P < 0.05; ++P < 0.01; +++P < 0.001

Lead contamination induced a significant increase in DNA and RNA contents in the kidney of lactating female rats compared with control animals (*P < 0.05*) (Figurey 1, 2(Fig. 1)(Fig. 2)). Conversely, an increase in DNA and mRNA contents was found in both male and female neonates following spirulina supplementation to the diet of lead-treated mothers.

### Renal lipid peroxidation, protein oxidation and H_2_O_2_ levels 

Our results demonstrated that lipid peroxidation products in the experimental groups varied greatly depending on mother treatment (Table 5(Tab. 5)). Lead intoxication resulted in a 78 % increase of TBARS concentration in kidney tissues of neonates compared to control. Similarly, a remarkable rise in AOPP, PCO and H_2_O_2_ levels in the kidney was also evident in the Pb group by 100 fold; +100 and +230 % and 86.36 %, 97.77 % and 138 fold in male and female neonates, respectively, when compared with controls (Table 5(Tab. 5)). That effect was even more remarkable in male compared to females. The supplementation of spirulina to the Pb-treated group, ameliorated all parameters cited above (*P <0.05*).

### Renal enzymatic and non-enzymatic antioxidants

Renal SOD activity of male and female offspring from mothers contaminated with lead decreased (by 47.56 % and 49.94 %, respectively) as compared to control animals (Table 5(Tab. 5)). This feature was significantly increased owing to the addition of spirulina to the mothers' diet (*P < 0.05*). 

Similar observations could be made concerning CAT activity in the kidney of neonates, which decreased markedly (by 60 % and 56.30 % in males and females, respectively) following Pb intoxication of mothers (Table 5(Tab. 5)). The addition of spirulina to the diet of mothers maintained control level of CAT activity in its offspring, although that effect was of less importance in female pups.

Considering lead intoxication of female rats, a 34-and 25 % decrease in GPX activity was observed in the kidney of male and female neonates, respectively. Such effect was less marked in female pups aged 14 days compared to male animals (*P < 0.05*). Here again, the addition of spirulina to the mothers'diet allowed GPx activity in newborn to remain close to control values (Table 5(Tab. 5)).

Moreover, GSH content in the kidney of neonate decreased following Pb intoxication of mothers (male: −33.63 %, *p < 0.05*; female: −34.19 %, *p < 0.05*) compared to controls. A significant recovery in kidney GSH content was observed in Pb + spirulina compared to lead treated group. Spirulina supplementation to the normal diet of control mothers had no effect *per se* on enzyme and non-enzymatic antioxidant activities in newborns (data not shown).

### Effect of Spirulina on histopathological examination of kidney tissue

**Figure 3:**
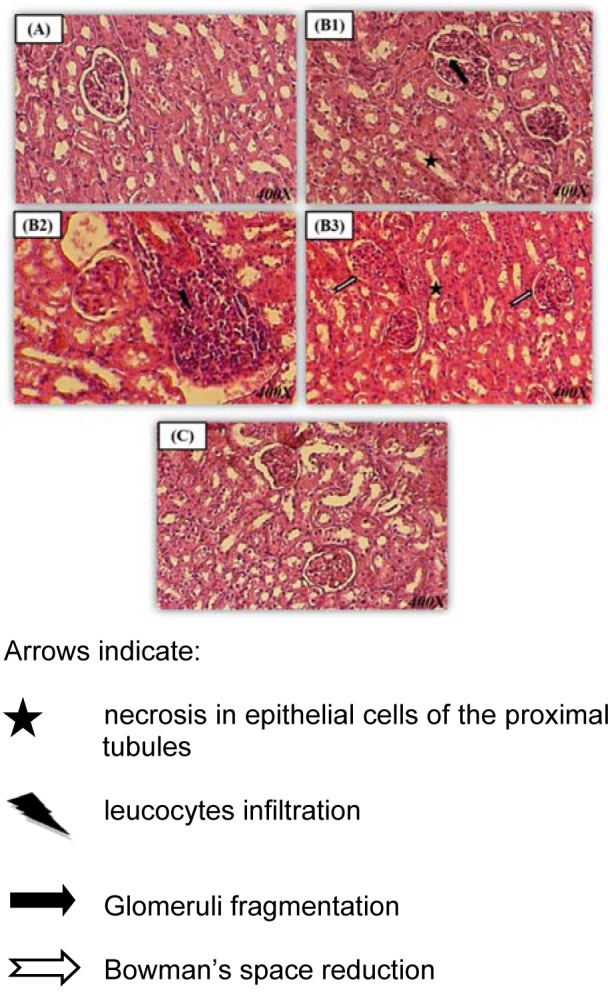
Kidney paraffin section photograph(s) of the 14 day-old newborn rat controls and experimental groups, controls (A), lead (B1, B2, B3) and spirulina Pb (C) presenting changes of the histopathology Stained with Hematoxylin-eosin (*Gr × 400)*.

Histological studies showed that control rats presented a normal kidney histoarchitecture with normal tubules and intact glomeruli (Figure 3A(Fig. 3)).

In the Pb-treated rats, kidney histological pictures showed numerous abnormalities detected in glomeruli and in convoluted tubules when compared to controls. In fact, kidney has a narrowed Bowman's space and fragmentation inside glomeruli (Figure 3B1, B3(Fig. 3)). A necrosis of the epithelial cells lining the tubules (Figure 3 B1, B3(Fig. 3)), associated with inflammatory leucocytes infiltration (Figure 3B2(Fig. 3)) were also observed in the kidney of lead-treated rats. 

Co-administration of Sp to Pb-treated rats enhanced the renal picture (Figure 3C(Fig. 3)). The histological pattern was normal in rats treated only with spirulina (data not shown). No significant differences were found between the control and spirulina groups, both of which showed normal glomeruli and tubules (Table 6(Tab. 6)).

## Discussion

Lead is a widespread pollutant of the environment. Its toxicity has been recognized as a major worldwide environmental health hazard that affects both animals and humans, especially pregnant ladies, infants and young children for a long time (Lalith and Muralidhara, 2014[43]). Lead's toxicity impacts depend on age, sex, route and duration of exposure level of intake.

Teratological studies have shown that malnutrition can cause several changes in rat's pups, like weight loss and growth impairment (Barkur and Bairy, 2015[16]). This aspect can be confirmed, as the regimen of exposure of female rats to Pb during late pregnancy and early postnatal periods, in the present study to have caused effect on the body weight and daily weight of their suckling pups. Moreover, mother and foetus can be considered as a unique system that remains in equilibrium through pregnancy. In fact, during pregnancy, lead crosses the placenta as there is no placental barrier for a heavy metal like lead (Bellinger et al., 1991[18]) and it accumulates in the foetus producing very undesirable toxic effects in the hematic system and affecting all the body organs (Markowitz, 2011[46]).

Accumulation of lead in blood makes them highly susceptible to its toxic action, such as covalent binding to proteins, interaction with stereospecific sites for divalent actions, generation of toxic metabolites, and oxidative damage (Aguilar-Dorado et al., 2014[7]). In the present study, abnormalities in some blood cell parameters of Pb treated newborn-rats were noted: the significant decrease in the number of red blood cells, in the Hb level and in the percentage of Ht, indicates the occurrence of microcytic anemia. Our results are consistent with a previous study of Ibrahim et al. (2012[38]), which showed the reduction of Hb confirmed the decrease in RBCs which may be attributed to the toxicity of lead acetate. Consequently, this reduction may be related to inhibition of erythropoiesis, leading to fatal conditions. Also, Pb treated mother rats induced a significant increase in WBC counts in their offspring, which can indicate the activation of immune system, consistent with another report (Ozsoy et al., 2013[50]).

However, the present study revealed that, SP supplementation induced a significant increase in both body weight and daily weight gain of mothers and offspring. Our results are consistent with many previous studies of SP in rats, ruminant and non-ruminant animals (Gargouri et al., 2016[34]; Abdel-Daim, 2014[2]). In addition, treatment with SP along with Pb normalized blood parameters to the control level. The beneficial effects of spirulina supplementation on growth and health were owing essentially to its protein richness as well as essential amino acids, fatty acids, minerals, vitamins, carotenoids and other antioxidant active (Sánchez et al., 2003[57]).

These hematological abnormalities cited in our study are the result of renal cell injuries. Indeed, blood distributes lead to tissues of the human body, especially to kidneys (ATSDR, 2007[11]). Pb-induced renal malfunction can be classified as acute and chronic nephropathy, inducing morphological changes like kidney weights. According to our findings, kidneys of the pups from lead-intoxicated mothers, during gestation, were significantly smaller than controls, which are in line with previous studies (Skröder et al., 2016[61]). However, both studies found an association between lead exposure and variation of kidney size.

According to the found data, kidney is the main site of biotransformation and elimination of xenobiotics (Soudani et al., 2010[62]). Exposure to lead caused alteration of renal function. Indeed, creatinine, urea and blood urea nitrogen (BUN) are used as sensitive biochemical markers employed in the diagnosis of renal damage. Our results showed an increase of plasma levels in creatinine, urea and BUN of the pups from lead-intoxicated mothers, may be due to kidney malfunction with a reduction in glomerular filtration rate, as shown by Soudani et al. (2010[62]); using another heavy metal. Indeed, experiments on mammals have shown that catabolism and/or the conversion of ammonia to urea leads to blood urea elevation which is related to arginase enzyme synthesis (El-Demerdash et al., 2004[24]). Similarly plasma creatinine increases in renal diseases gave prognostic importance than those of other nitrogenous substances (Amin et al., 2010[10]). Uric acid, used in the present work is known for its free radicals scavenger capacity (Alvarez-Lario and Macarron-Vicente, 2010[9]). In fact, our results showed a decrease in plasmatic uric acid levels which may be associated to multiple biological effects such as endothelial malfunction, platelet aggregation, increased oxidative stress, and high levels of inflammatory markers (Harlalka et al., 2007[36]). Furthermore, we observed that the disturbance of urine biochemical marker in the Pb-treated group could be explained by the severe and irrevocable morphological and functional changes, such as glomerular and tubule interstitial changes attributed to the generation of oxidative stress, accompanied by hyperuricemia, vacuolization and renal breakdown (Navarro-Moreno et al., 2009[48]).

The supplementation of spirulina to the diet of lead treated rats during gestation and lactation attenuated the kidney impairment in neonate as suggested by a significant amelioration of the renal biomarkers indicated earlier. This is owing to the accelerated regeneration of the extent of tubular malfunction under the influence of biliprotein pigment known as phycocyanin drug that can be found in spirulina and that exerted probably a diuretic activity (Farooq et al., 2004[28]). Besides, according to Abdel-Daim (2014[2]) this alga is rich in potassium, which is reported to have a diuretic effect. In addition, the presence of flavonoids in spirulina alga can explain the increase of diuresis as reported by Yuliana et al. (2009[67]) who have indicated that flavonoids cause polyuria.

The present study is corroborated by the observations of Abdel-Daim (2014[2]) who reported that spirulina protects adult mice from deltamethrin-induced nephrotoxicity through its antioxidant properties. Furthermore LDH, a potent marker of oxidative stress, is liberated in the blood stream after cell membrane integrity disruption (Zou et al., 2001[68]). Our studies showed that, in lead-treated rats, LDH activity decreased significantly in kidney tissues while it increased in plasma when compared with controls. These changes are related to lead-administration during early postnatal period.

Also, the current study demonstrated a significant decline of ALP activity in the plasma in newborns which were exposed to lead. This decrease can be attributed to a decreased osteoblastic activity in bone, since ALP is formed in the osteoblasts. Our results showed also a significant reduction in plasma GGT activity in Pb-treated rats which is due to the occurrence of oxidative stress through an excessive generation of ROS, as reported by Abdelhalim and Moussa (2013[4]) in rats exposed to gamma radiation. These findings are approved by Farooq et al. (2004[28]), who suggested that enhanced plasmatic enzyme markers excretion (ALP and GGT) in urolithic animals, is associated with the retention and deposition of crystals in the kidney. These perturbations are related to the damage of the brush border membrane of the renal tubules.

However, administration of spirulina to the diet of lead treated rats during gestation and lactation in the drug of pre-treated, ameliorated a marker enzymes. This correction is owing to the presence of the nephroprotective agent such as phycocyanin (Romay et al., 1999[55]).

Many of the newest researches on lead toxicity focused on its effects like inducing oxidative stress. The reactive species produced in oxidative stress can cause direct damage of the DNA and are therefore mutagenic. However, appropriate data on the oxidative DNA damage indirectly induced by lead in the kidney of animals, during prenatal exposure, are not provided. In the current study, the kidney DNA and RNA levels in the offspring dramatically increased as a result of lead intoxication. Our results are in agreement with other studies previously reported in adult animals (Valverde et al., 2001[64]; Azqueta et al., 2009[15]), who have corroborated the big relation between DNA occurence damage and ROS or ROS derived lipid-hydro peroxide production. In addition, lead-induced elevated generation of TBARS in the offspring as mentioned in our study and as explained previously by recent investigations performed on mice (Sharma and Singh, 2014[59]). This TBARS elevation was explained via excessive production of ROS; consequently, subsequent loss of membrane integrity and inactivation of tubular cell constituents. 

The inexorable generation of ROS during lead exposure was, also, correlated with the significantly elevated levels of protein damage, including PCO (early marker of protein oxidation) and AOPP levels. Under our experimental conditions and for the first time, the increased PCO and AOPP levels were detected in the kidney of offspring of lead-poisoned mothers. The increase of proteins can be related to carbonyl group formation into side chains and/or reduction of sulfhydryl groups in susceptible amino acids following a protein modification, by direct attack of ROS (Sharma and Singh, 2014[59]).

Defence of renal cells against oxidative stress is maintained by several mechanisms including antioxidant status. Oral treatment of Pb exposure in experimental studies induced significant decrease in the activity of antioxidant enzymes SOD, CAT, GPx, and GSH in renal tissues obtained in the current study. According to Abdel Moneim et al. (2011[1]) and Farmand et al. (2005[27]), the decrease of antioxidant activity may be explained by the complexity of the effects of lead on these enzymes, since Pb can competitively inhibit bio elements absorption and/or replace them in the active sites of enzymes or bind to -SH groups of proteins.

Interestingly, co-treatment with spirulina was very effective in the prevention of antioxidant status in the kidney of newborns exposed to lead, objectified by a significant increase of enzymatic (SOD, CAT and GPx) and non-enzymatic (GSH) activities. This could be explained by richness content of antioxidant active ingredients, such as C-phycocyanin, ß-carotene, minerals, vitamins, etc. (Avdagic et al., 2008[12]). In other studies of renal injury, SP induced significant renoprotective indicating a potential therapeutic role of SP against gentamicin, cisplatin, cyclosporine and Deltamethrin-induced nephrotoxicity and ROS production (Abdel-Daim et al., 2013[3]; Avdagic et al., 2008[12]; Mohan et al., 2006[47]).

Biochemical results, obtained in the current study, for the first time, were substantiated by histological examination. In fact, the histo-architecture of the newborn's kidney that was treated with lead shows an infiltration of leucocytes in tissue, a renal tubular damage characterized by necrosis of the epithelial cells lining the tubules. There were also an enlarged Bowman space and glomeruli fragmentation. The supplementation of spirulina to the Pb treated newborn's rats ameliorated significantly the histological alterations and the physiological state thanks to its radical scavenging properties and its regulatoral effect on the antioxidative systems (Abdel-Daim et al., 2013[3]).

## Conclusion

In summary, our data, for the first time, showed that lead intoxication of pregnant and lactating mother's causes dramatic kidney damages in their offspring. This nephrotoxicity induced by lead led to kidney injury caused by ROS generation. However, the consumption of spirulina by mother rats provided a near complete protection against the adverse effects of Pb on neonate kidney through its active constituents such us phycocyanin, vitamins, minerals and polyphenols. Further study on human subjects is now needed to confirm its potential.

However, the consumption of spirulina by mother rats provided a near complete protection against neonate kidney Pb effects. This effect is due to alga's active constituents such us phycocyanin, vitamins, minerals and polyphenols. Further studies to be done on human subjects are actually necessary to confirm the spirulina potential effects.

## Acknowledgements

This work was supported by DGRST grant (Appui à la Recherche Universitaire de base UR/13 ES-73). The authors are grateful to Prof. N. Khedimi, ESP teacher at the Faculty of Sciences of Gafsa, for proof reading the manuscript and Dr Xavier Dauvergne for data analyses.

## Conflict of interest statement

The authors report no conflicts of interest associated with this manuscript.

## Figures and Tables

**Table 1:**
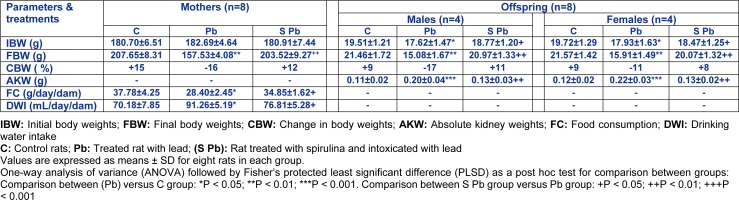
Effects of lead exposure and/or spirulina on mother rats and their offspring ((C), (Pb) and (S Pb)) on their Initial and final body, absolute kidney weights, daily food and water consumption from day 5 of pregnancy to day 14 after delivery

**Table 2:**
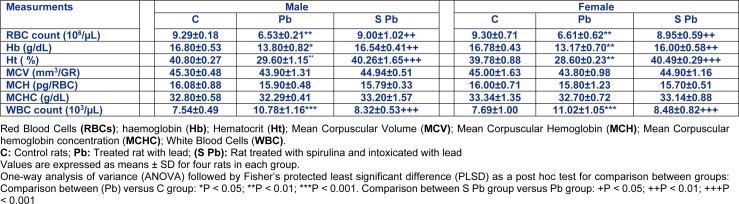
Effects of lead exposure and/or spirulina on 14 day-old rats ((C), (Pb) and (S Pb)) on hematological parameters

**Table 3:**
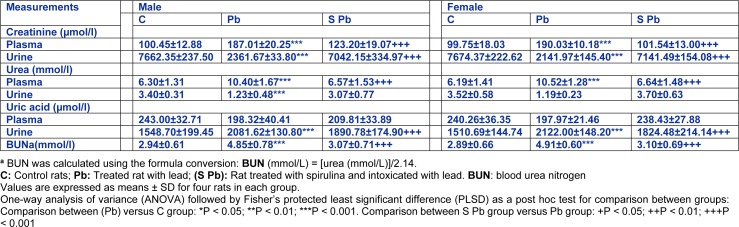
Effects of lead exposure and/or spirulina on 14 day-old rats ((C), (Pb) and (S Pb)) on plasma and urinary levels of creatinine, urea and uric acid, and BUN. BUN: blood urea nitrogen

**Table 4:**
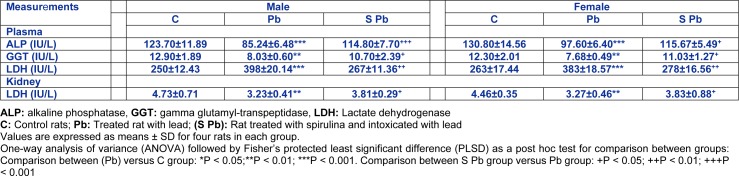
Effects of lead exposure and/or spirulina on the 14 day-old rats ((C), (Pb) and (S Pb)) on plasma ALP, GGT and LDH activities and kidney LDH activity

**Table 5:**
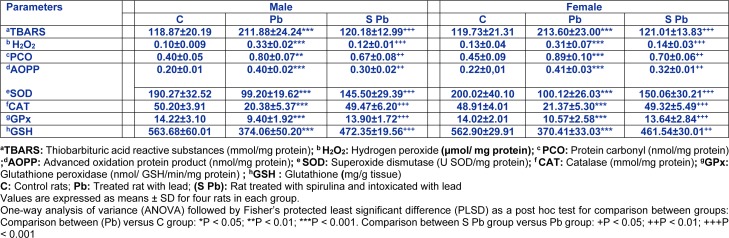
Effects of lead exposure and/or spirulina on the 14 day-old rats ((C), (Pb) and (S Pb)) on renal TBARS, H_2_O_2_, PCO, AOPP levels and activities of CAT, SOD, GPx and GSH

**Table 6:**
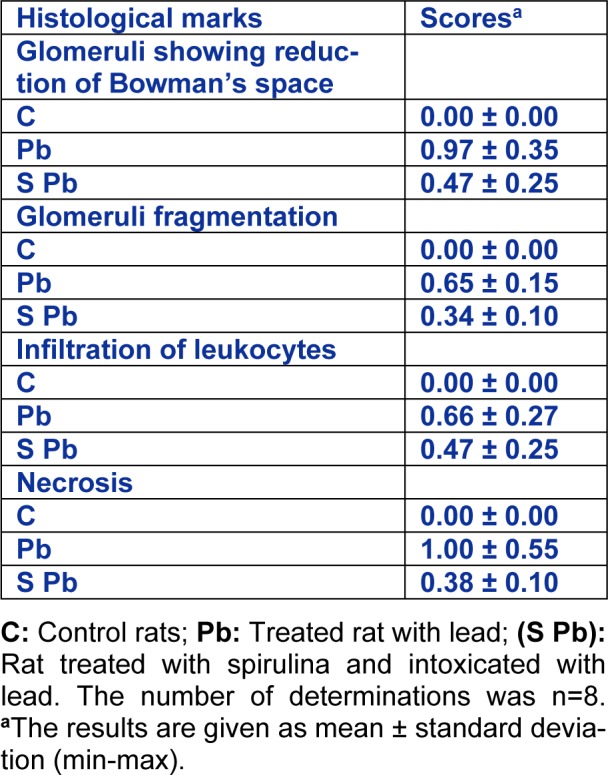
Renal histopathological analysis in suckling rat
